# Uncommon musculoskeletal femoral hydatid cyst disturbs a female for a year: Case report

**DOI:** 10.1016/j.amsu.2020.04.025

**Published:** 2020-05-11

**Authors:** Aya Zazo, Rama Zazo, Mohammad Nour Shashaa, Mohamad Shadi Alkarrash, Muhamad Zakaria Brimo Alsaman, Ammar Niazi

**Affiliations:** a Faculty of Medicine, University of Aleppo, Aleppo, Syria; b General Surgery Department, Faculty of Medicine, Aleppo University Hospital, University of Aleppo, Aleppo, Syria

**Keywords:** Hydatid, Cyst, Echinococcosis, Thigh, Muscle

## Abstract

“Hydatid cyst” which also known as cystic Echinococcosis is a parasitic infestation caused by the larval stage of Echinococcus granulosus. The liver and lungs are the most sites to occur. Incidence in muscles is exceptionally rare.

We report a case of a 36-year-old female presented with an uncomfortable mass in the upper medial of her right thigh without any presence of other symptoms. She lived in a rural area in Manbij, which is an endemic area of hydatid cysts in Syria. She was a shepherdess; therefore she had direct contact with sheep and dogs. Ultrasound examination showed a cyst located between adductor longus muscle and gracilis muscle closed to the deep femoral artery. The patient was treated with pharmaceutical therapy for a week before cystectomy, which was done under general anesthesia. The cyst was dissected between the fibers of adductor longus muscle from the lateral side and fibers of the gracilis muscle from the medial side. The cyst with all its layers was resected.

Musculoskeletal Echinococcosis is a rare disease, because of intramuscular growth of cysts is restricted by muscle's contractility, the muscles are undesirable habitat for Echinococcus granulosus and because of the hepatic barrier role. Many cysts are revealed by complications such as nerve compressions, infections simulating an acute abscess or a malignant tumor.

Hydatid cyst present as mass of soft tissue, particularly in endemic areas, as a result of contaminated water. MRI considered the best technique in the diagnosis.

## Highlights

• Incidence of Hydatid cyst in muscles is exceptionally rare.
• MRI considered the best technique in the diagnosis.
• Confirming the final preoperative diagnosis of muscle hydatid cysts is important.
• Hydatid cyst should consider as a differential diagnosis for masses of soft tissue.

## 1 Introduction

Hydatid cyst which also known as cystic echinococcosis is a parasitic infestation caused by the larval stage of Echinococcus granulosus [1].

The highest incidence is in the countries that raise sheep, cattles, and dogs, especially in the Middle East, Central Europe, Australia, South America, and the Mediterranean basin. The liver and lungs are frequently the most infected sites; the muscles are rarely infected and count about 2.2% [2].

The presence of the hydatid cyst in the thigh is rare and counts about 0.35%–15% [3,4].

Human Hydatid disease is a zoonotic infection. Humans are infected by consumption food and water contaminated with Echinococcus's eggs [2].

These cysts manifest as slow-growing masses of soft tissue and can be associated with fistulization and signs of inflammation [5,6].

The clinical manifestation is insidious and nonspecific, frequently causing tardiness in diagnosis. The nonspecific clinical symptoms can be concise as a painless non-inflammatory tumescence increases progressively in size over many years while maintaining the patient's good general health. However, a particular number of cysts are revealed by complications such as nerve compressions or infections simulating an acute abscess or a malignant tumor [6].

Here we present a rare case of hydatid cyst located between adductor longus muscle and gracilis muscle of a 36-year-old female.

The work in this case report has been reported in line with the SCARE 2018 criteria [7].

## 2 Case presentation

**Clinical findings:** A 36-year-old female with a body mass index (BMI) of 26, 4 kg/m2, presented to the surgical clinic with a one-year history of an uncomfortable mass in the upper medial aspect of her right thigh. The patient suffered from heaviness in the thigh without any presence of other symptoms. She lived in a rural area in Manbij, which is an endemic area of hydatid cysts in Syria. She was a shepherdess; therefore she had direct contact with sheep and dogs. The patient had no family, surgical, or medical history. Physical examination showed an unpainful mobile mass in the upper medial aspect of the thigh without any features of acute inflammation or bruise. No erythema or any sign of lymphadenopathy.

**Fig. 1:**
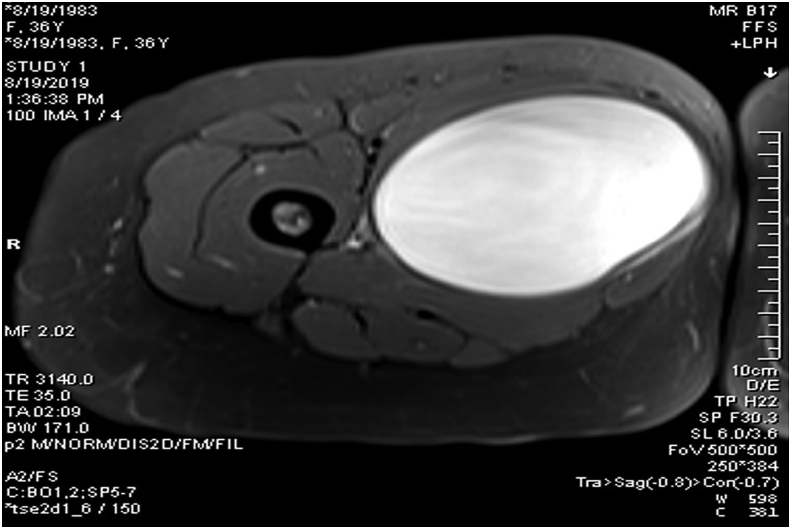
Transverse plane by MRI scan shows a cystic mass in the right thigh closed to the deep femoral artery, it was characterized by a low single intensity in T1-weighted scan and high signal intensity in T2-weighted scan.

**Diagnostic assessment:** Laboratory tests, including serum chemistries, complete blood cell count, erythrocyte sedimentation rate (ESR), coagulation profile, and C - reactive protein (CRP) showed normal results. Weinberg test was carried out, and it was negative. Initially, an ultrasound examination “US” was performed, which showed a double-wall cyst with pure liquid in adductor longus muscle and gracilis muscle closed to the deep femoral artery. Then magnetic resonance imaging (MRI) was performed, which showed a cystic mass approximately 124× 80 × 110 mm closed to the deep femoral artery [Fig. 1]. The cyst was characterized by a low single intensity in T1-weighted scan and high signal intensity in T2-weighted scan. Depending on the history of the patient (sheep farmer), Physical examination and radiological manifestations, a diagnosis of a hydatid cyst was made. More imaging studies were done to confirm the diagnosis and discover other possible sites of involvement such as lung and liver. A computed tomography scan (CT) scan for the chest and abdomen showed no other sites of involvement.

**Fig. 2:**
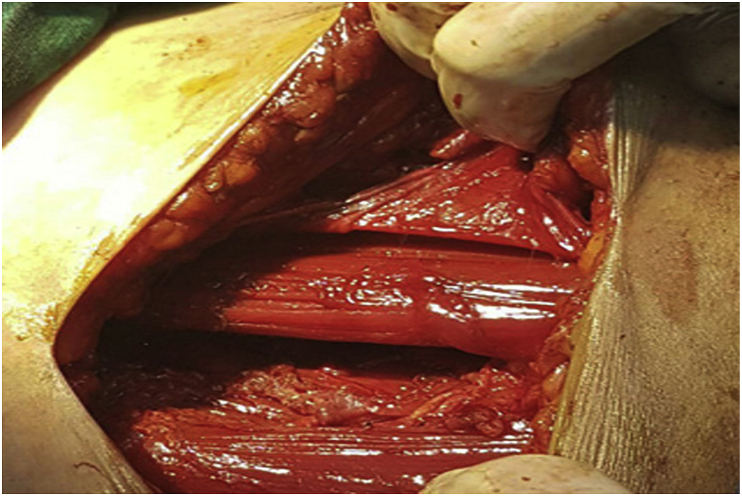
Fibers of adductor longus muscle from the lateral side, and fibers of gracilis muscle from the medial side.

**Fig. 3:**
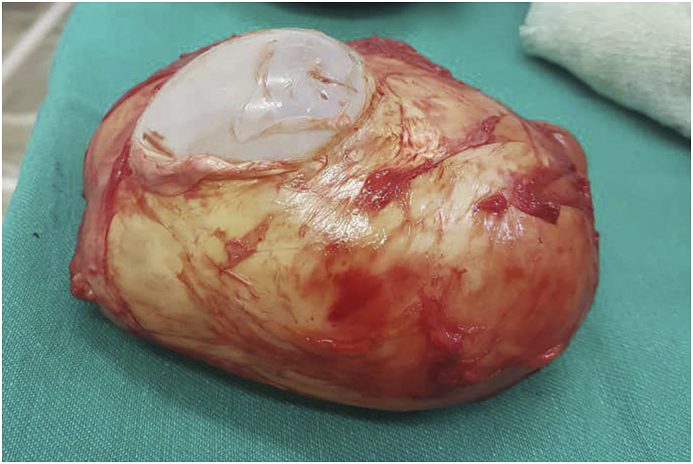
The cyst after removal with all its layers.

**Therapeutic Intervention:** The patient was treated with Albendazole (300) mg for a week before surgery was done. Cystectomy was performed under general anesthesia. A medial longitudinal incision was done, and the deep femoral artery was isolated. The cyst was injected with a stramid 15% (cetrimide–chlorhexidine combination) to reduce the risk of recurrence, after that, the cyst was dissected between the fibers of adductor longus muscle from the lateral side and fibers of gracilis muscle from the medial side [Fig. 2]. The cyst with all its layers was resected without any rupture or puncture [Fig. 3]. The patient received postoperative pharmacological therapy with Albendazole (300) mg for three months.

**Follow-up and outcomes:** Postoperatively, the patient was followed up for a month without any recurrence manifestations.

## 3 Discussion

Hydatid disease which also called cystic Echinococcosis is a parasitic disease caused by the larval level of Echinococcus [1].

There are two species of Echinococcus “E. Granulosus and E. Multilocularis” cause cystic Echinococcosis and alveolar Echinococcosis in humans. This disease prevails principally in Asia, North and East Africa, South America, Australia, and the Middle East. The most common sites of echinococcal cysts are in the liver (62%) and lungs (20%) but can affect any part of the body [2].

Differential diagnosis of hydatid disease should be considered for every soft cystic mass in any anatomical location, especially in endemic areas of the disease [8].

Musculoskeletal Echinococcosis is an infrequent case counting for only 3–5% of all cases. Many factors explain the rare occurrence of muscular participation: First, the muscle is classified as an undesirable habitat for the Echinococcus because of the high levels of lactic acid, which makes the muscle an inconvenient growth environment. Second, the intramuscular growth of cysts is restrained by the muscle's contractility. Third, the consideration of the hepatic barrier role, where the implantations at this site requires a passage through the filters of the liver and lung [5,9].

Computed tomography has superiority over ultrasound because of having better documentation of site, size, and structure of the cyst. The differentiating sign of hydatid disease may show on MRI as an intensive rim [10].

To confirm the diagnosis, several popular tests are usually applied, such as Sero-logic tests, indirect immunofluorescence antibody test, ELISA, immunoelectrophoresis, and immunoblot test [1].

Weinberg test or Complement Fixation test (CF) is one of the serological methods commonly used to diagnose the hydatid disease, the sensitivity of CF test ranges from 36% to 93%, This method also has a prominent role for postoperative evaluation [11].

In general, MRI has a higher specificity and sensibility than ultrasound for hepatic hydatid cyst [12].

It also allows a better characterization of anatomical relations and helps surgical management of cyst. It is essential to confirm the final preoperative diagnosis of skeletal muscle hydatid cysts. This contraindicates confirm treatment options like marginal excision or incisional biopsy due to the likelihood of dissemination and anaphylactic shock on spillage [13].

Pericystectomy remains the treatment of choice in musculoskeletal hydatid cysts. Percutaneous aspiration, infusion of scolicidal agents like chlorhexidine gluconate, and re-aspiration PAIR, under imaging ultrasound or CT guidance, can be used as an alternative to surgery in inoperable cases [14].

Supplementary chemotherapy with anthelmintic for skeletal muscle hydatid disease is debatable, and currently, no evidence provides sufficient backing on the benefit of its association with conservative treatment. Albendazole remains the gold standard drug administered in adjuvant therapy [15]. However, In general, MRI is considered the best procedure in diagnosing a Hydatid cyst. Eventually, the Hydatid cyst should be considered as a differential diagnosis for any mass of soft tissues, especially in endemic regions.

## 4 Conclusion

Hydatid cyst incidence is extremely rare in muscle, and most occur in the liver, lungs, or both. The disease is zoonotic, the result of contaminated water, via Echinococcus granulosus. Hydatid cyst should be considered as a differential diagnosis for masses of soft tissue, especially in endemic areas. MRI considered the best technique in the diagnosis.

## Patient perspective

The patient participated in the treatment decision and she was satisfied with the results of the treatment. Her perspective on this treatment was to get rid of the uncomfortable mass without complications.

## Informed consent

Written informed consent was obtained from the patient for publication of this case report and accompanying images. A copy of the written consent is available for review by the Editor-in-Chief of this journal on request.

## Provenance and peer review

Not commissioned, externally peer-reviewed.

## Ethical approval

Not required for case reports at our hospital. Single case reports are exempt from ethical approval in our institution.

## Sources of funding

There are no sources of funding.

## Author contribution

Aya Zazo: data collection, revising critically, wrote the manuscript.

Rama Zazo: patient care, revision, corresponding author.

Mohammad Nour Shashaa: review and editing, data analysis, wrote the manuscript.

Mohamad shadi Alkarrash: design of the study, revision, validation.

Muhamad Zakaria Brimo Alsaman: patient care, data collection, wrote the manuscript.

Ammar Niazi: managed the patient and did the surgery, the supervisor, patient care, revising critically.

All authors read and approved the final manuscript.

## Trial registry number

None.

## Guarantor

Dr.Ammar Niazi.

## Consent

Written informed consent was obtained from the patient for publication of this case report and accompanying images. A copy of the written consent is available for review by the Editor-in-Chief of this journal on request.

## Declaration of competing interest

The authors declare that they have no conflict of interest.
