# Supplementary material for: Uncommon musculoskeletal femoral hydatid cyst disturbs a female for a year: Case report
Source: Ann Med Surg (Lond). 2020 May 11;55:30–2. doi: 10.1016/j.amsu.2020.04.025 (PMC7231815; doi:10.1016/j.amsu.2020.04.025)
Supplement: Multimedia component 1 [file mmc1.docx]

| **SCARE 2018 Checklist** | | | |
| --- | --- | --- | --- |
| **Topic** | **Item** | **Checklist item description** | **Page Number** |
| **Title** | **1** | **Uncommon musculoskeletal femoral hydatid cyst disturbs a female for a year: case report** | 1 |
| **Key Words** | **2** | **Hydatid, Cyst,  Echinococcosis, Thigh, Case report.** | 1 |
| **Abstract** | **3a** | **Incidence of hydatid cyst in muscles is exceptionally rare.** | 1 |
|  | **3b** | **A 36-year-old female presented with uncomfortable mass in the upper medial of her right thigh without any presence of other symptoms. Ultrasound examination showed a cyst located between adductor longus muscle and gracilis muscle closed to the deep femoral artery.** |  |
|  | **3c** | **Ultrasound examination showed a cyst located between adductor longus muscle and gracilis muscle closed to the deep femoral artery. The patient was treated with pharmaceutical therapy for a week before cystectomy, which was done under general anesthesia. The cyst was dissected between the fibers of adductor longus muscle from a lateral side and fibers of gracilis muscle from a medial side. The cyst with all its layers was resected.** |  |
|  | **3d** | **Hydatid cyst present as masses of soft tissue, particularly in endemic areas, as a result of contaminated water. MRI considered the best technique in the diagnosis.** |  |
| **Introduction** | **4** | **The highest incidence is in the countries that raise sheep,cattle and dogs, especially in the Middle East, Central Europe, Australia, South America, and the Mediterranean basin. The liver and lungs are frequently the most infected sites; the muscles are rarely infected and count about 2.2 %. The presence of the hydatid cyst in the thigh is rare and counts about 0.35% to 15%. Human Hydatid disease is a zoonotic infection. Humans are infected by consumption food and water contaminated with Echinococcus's eggs. These cysts manifest as slow-growing masses of soft tissue and can be associated with fistulization and signs of inflammation.** | 1 |
| **Patient Information** | **5a** | **A 36-year-old female with a body mass index (BMI) of 26, 4 kg/m2.** | **2** |
|  | **5b** | **presented to the surgical clinic with a one-year history of an uncomfortable mass in the upper medial aspect of her right thigh. The patient suffered from heaviness in the thigh without any presence of other symptoms.** |  |
|  | **5c** | **The patient had no family, surgical, or medical history.** |  |
|  | **5d** | **She lived in a rural area** in Manbij, which is an endemic area of hydatid cysts in Syria. She was a shepherdess; therefore she had a direct contact with sheep and dogs**.** The patient had no family, surgical, or medical history. |  |
| **Clinical Findings** | **6** | **Physical examination showed an unpainful mobile mass in the upper medial aspect of the thigh without any features of acute inflammation or bruise. No erythema or signs of lymphadenopathy.** | 2 |
| **Timeline** | **7** | **For one year the patient complained of uncomfortable mass in the upper medial aspect of her right thigh. The patient suffered from heaviness in the thigh without any presence of other symptoms. She was diagnosed with hydatid cyst depending on US and MRI results immediately. The patient was treated with** Albendazole (300) mg for a week before surgery was done. **Cystectomy was performed under general anesthesia. The patient received postoperative pharmacological therapy with albendazole (300) mg for three months. Postoperatively, the patient was followed up for a month without any recurrence manifestations.** | 2 |
| **Diagnostic Assessment** | **8a** | **Physical examination showed an unpainful mobile mass in the upper medial aspect of the thigh without any features of acute inflammation or bruise. Laboratory tests showed normal results. (US) showed a double-wall cyst with pure liquid in adductor longus muscle and gracilis muscle closed to the deep femoral artery. Magnetic resonance imaging (MRI) showed cystic mass approximately 124 × 80 × 110 mm closed to the deep femoral artery. CT scan for the chest and abdomen showed no other sites of involvement. Weinberg test carried out, and it was negative.**  **ELISA test was not performed because it is not available in our hospital.** | 2 |
|  | **8b** | **Lack of diagnostic tools and high cost** |  |
|  | **8c** | **MRI results were suggestive of hydatid cyst.** |  |
|  | **8d** | **A computed tomography scan (CT) scan for the chest and abdomen showed no other sites of involvement. Weinberg test carried out, and it was negative.** |  |
| **Therapeutic Intervention** | **9a** | **pharmacological therapy,** Albendazole (300) mg | 2 |
|  | **9b** | **A diagnosis of hydatid cyst was made and The patient was treated with** Albendazole (300) mg **for a week before surgery was done.** . The cyst was injected with a stramid 15% to reduce the risk of recurrence. **Cystectomy was performed under general anesthesia** |  |
|  | **9c** | The cyst was injected with a stramid 15% to reduce the risk of recurrence. **Cystectomy was performed under general anesthesia where the cyst with all its layers was resected.** |  |
|  | **9d** | **general surgeon who** resected The cyst with all its layers **without any rupture or puncture** |  |
|  | **9e** | **There were not any changes in the interventions.** |  |
|  | **9f** | The patient received postoperative pharmacological therapy with Albendazole (300) mg for three months. |  |
| **Follow-up and**  **Outcomes** | **10a** | **The patient was followed up for a month without any recurrence manifestations.** | 2 |
|  | **10b** |  |  |
|  | **10c** | A computed tomography scan (CT) scan for the chest and abdomen showed no other sites of involvement |  |
|  | **10d** | **There were no complications** |  |
| **Discussion** | **11a** | **Strengths: Musculoskeletal Echinococcosis is infrequent case, and the intervention technique was unusual.**  **Weaknesses: the follow up was only for one month because of the Rural accommodation of the patient.** | 3 |
|  | **11b** | **According to guidelines The most common sites of echinococcal cysts are in the liver (62%) and lungs (20%). Our case is hydatid cyst located between adductor longus muscle and gracilis muscle.** |  |
|  | **11c** | **We mentioned causes, common symptoms and the best treatment briefly.** |  |
|  | **11d** | **Hydatid cyst should be considered as a differential diagnosis for masses of soft tissue, especially in endemic areas. MRI considered the best technique in the diagnosis.** |  |
|  | **11e** | **Hydatid cyst should be considered as a differential diagnosis for masses of soft tissue, especially in endemic areas. MRI considered the best technique in the diagnosis.** |  |
| **Patient Perspective** | **12** | **The patient participated in treatment decision and she was satisfied with the results of the treatment. Her** perspective on this treatment **was to get rid of the uncomfortable mass without complications** | 4 |
| **Informed Consent** | **13** | **Informed consent was obtained.** | 4 |
| **Additional Information** | **14** | **Conflicts of Interest:**  **The authors declare that they have no conflict of interest.**  **Sources of funding:**  **There are no sources of funding.**  **Ethical approval:**  **Not required for case reports at our hospital. Single case reports are exempt from ethical approval in our institution.** | 4 |
